# Relating gene expression data on two-component systems to functional annotations in *Escherichia coli*

**DOI:** 10.1186/1471-2105-9-294

**Published:** 2008-06-25

**Authors:** Anne M Denton, Jianfei Wu, Megan K Townsend, Preeti Sule, Birgit M Prüß

**Affiliations:** 1 Department of Computer Science and Operations Research, North Dakota State University, Fargo, ND 58105, USA; 2 Department of Veterinary and Microbiological Sciences, North Dakota State University, Fargo, ND 58105, USA

## Abstract

**Background:**

Obtaining physiological insights from microarray experiments requires computational techniques that relate gene expression data to functional information. Traditionally, this has been done in two consecutive steps. The first step identifies important genes through clustering or statistical techniques, while the second step assigns biological functions to the identified groups. Recently, techniques have been developed that identify such relationships in a single step.

**Results:**

We have developed an algorithm that relates patterns of gene expression in a set of microarray experiments to functional groups in one step. Our only assumption is that patterns co-occur frequently. The effectiveness of the algorithm is demonstrated as part of a study of regulation by two-component systems in *Escherichia coli*. The significance of the relationships between expression data and functional annotations is evaluated based on density histograms that are constructed using product similarity among expression vectors. We present a biological analysis of three of the resulting functional groups of proteins, develop hypotheses for further biological studies, and test one of these hypotheses experimentally. A comparison with other algorithms and a different data set is presented.

**Conclusion:**

Our new algorithm is able to find interesting and biologically meaningful relationships, not found by other algorithms, in previously analyzed data sets. Scaling of the algorithm to large data sets can be achieved based on a theoretical model.

## Background

Microarray experiments are popular tools in functional genomics. Correspondingly, many techniques have been developed to analyze their results. Typical questions asked include which genes are differentially expressed [1], and which groups of genes show similar expression in multiple related experiments [2]. Identifying functional patterns among the resulting list or groups of genes is a separate step that is not supported by standard clustering techniques. Biclustering techniques have been developed to group functions and experiments simultaneously [3,4]. Gene expression information is also used to predict gene functions [5]. In experiments related to transcriptional regulation, the objective is to understand the regulation process rather than predicting protein function. That means that predictive techniques are not appropriate.

Recently, gene set enrichment analysis, GSEA [6,7], has become a popular tool for relating expression values to properties that define sets of genes. Gene set analysis conventionally tests the relationship of gene expression experiments related to a discrete phenotype to any one of a number of possible grouping criteria. A phenotype may distinguish between healthy and diseased tissue or between different strains of bacteria. GSEA also enables such analysis for continuous phenotype labels. This feature can be used for time series gene expression data. However, this analysis type requires knowing what the profile of interest is. Two example applications are suggested in the GSEA documentation [8]: An expected profile such as a peak or alternatively the expression of a particular gene may be used as profile of interest.

**Figure 1:**
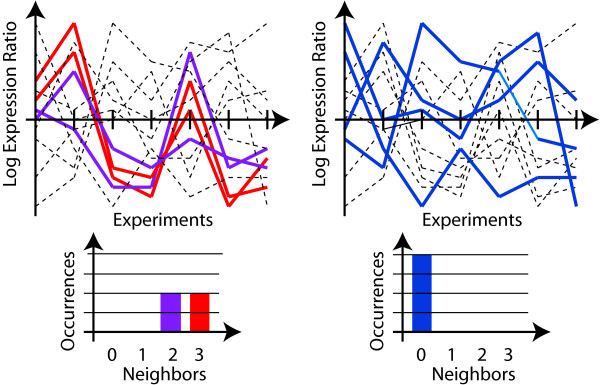
**Sample expression profiles for two subsets of data**. The top panels show gene expression profiles over multiple related experiments. The same set of curves are shown in the left and in the right panel. In each panel, a different subset of profiles is highlighted, corresponding to genes of a different functional designation. The bottom panels quantify the presence of patterns by identifying neighboring relationships among profiles using a product similarity measure. The number of neighbors, for all genes that show the function, is summarized in a histogram.

Our algorithm does not require any input of an expected profile. The distribution of gene expression profiles alone is what determines whether the gene set shows enrichment. This allows us to not only consider time course experiments for which a particular profile may be natural to expect but any group of related experiments. Fig. 1 illustrates the concept: The same set of curves is shown in the left and in the right panel of the figure. Each curve represents a gene expression profile over multiple related experiments. In each panel, a different subset of profiles is highlighted, corresponding to genes of a different functional designation. The highlighted profiles in the left panel show a clear pattern, while the ones in the right panel do not. We quantify the presence of patterns by identifying neighboring relationships among profiles using a product measure. If a gene has many neighbors with a similar expression profile – more than would be expected by random chance – then it supports the existence of a pattern. The number of neighbors, for all genes that show the function, is summarized in a histogram. In the left panel, two of the profiles have all other three genes as neighbors, and the others have two neighbors. In the right panel, in contrast, none of the profiles has any neighbor. It is expected that some profiles may have neighbors by random chance alone. For this reason, we compare the resulting histograms with ones that are generated for random subsets of the same size.

**Figure 2:**
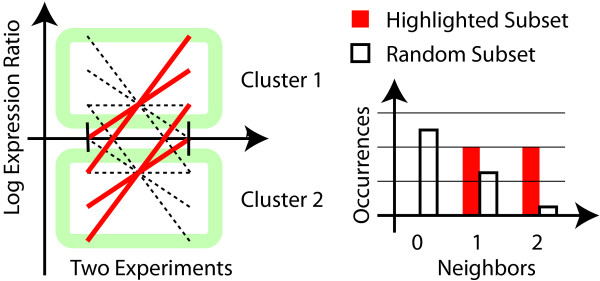
**Toy example to illustrate limitations of a two-step approach**. The schematic demonstrates why clustering or biclustering followed by gene-enrichment analysis may miss patterns that can be found by our approach. The left panel shows two hypothetical experiments side by side using the same type of visualization as in Fig. 1. Genes with a particular functional annotation are highlighted; others are represented by dashed lines. It can be seen that the natural clusters in the data set do not show enrichment and would, hence not be considered significant by a two-step approach. In contrast, for our approach the histogram of neighbors (right panel) differs from what would be expected for a random subset.

The concept of gene set enrichment has previously been applied to the results of clustering or biclustering [9]. In this approach it is tested whether any one particular functional category shows enrichment for the clustering that was determined. Fig. 2 illustrates the limitations of such a two-step process. This schematic uses the same type of representation as Fig. 1, in which experiments are shown side-by-side and are connected by lines. The example was constructed such, that there are two clear clusters that would be identified by most clustering algorithms. One cluster is formed by the five genes that have primarily positive expression values and the other cluster by those that have primarily negative values. Neither of these clusters shows enrichment for the function that is highlighted, since each of them has two out of five members with the annotation. The genes with the annotation, nevertheless, show a clear pattern. The right panel illustrates that the histogram of neighbors for the subset of highlighted genes differs from what would be expected for a random subset, showing that such patterns are accessible to our algorithm. Notice that the similarity among the highlighted genes is not sufficient to consider them as a cluster based on expression values alone. Our algorithm, in contrast, tests solely whether their distribution within the space of gene expression values differs from what would be expected. No information is lost prior to that test. If clustering/biclustering is performed first, the information that the highlighted genes are similar despite being in different clusters is lost. This limitation of the clustering-based approach cannot be resolved by using different clustering algorithms that use functional information [10] since such algorithms would distort the probability of finding enrichment. Our one-step approach resolves the problems of the two-step approaches at a fundamental level.

Work by Kim *et al. *[11] can also be compared with our approach. In this work conventional statistical techniques are used, in particular the Pearson correlation coefficient. For that reason, gene sets have to be grouped into functional clusters first, such that there is a sufficiently large number of genes in each group. The histogram-based analysis in our presented algorithm can be applied to fewer genes, and furthermore performs a direct comparison with a randomized distribution. Using the Pearson correlation coefficient directly as a measure of coherence amounts to an assumption of a homogeneous comparison distribution, much as our theoretical model, which is introduced as a high-performing alternative. In contrast to the work by Kim and coworkers, our algorithm allows a more accurate comparison based on resampling. Other modifications have been proposed to the gene set enrichment concept, such as dimensionality reduction [12] and considering multiple functional groups [13]. Reviews of some related techniques can be found in [14,15].

Our algorithm is tested on a published set of microarray data [16], in which each experiment corresponds to one knock-out mutant that represents one two-component system, compared to wild-type *E. coli*. Two-component systems are regulatory systems that involve two protein components, a histidine kinase and a response regulator. In response to an environmental stimulus, the histidine kinase phosphorylates itself and then transfers the phosphate to the response regulator. Transcription regulation only happens when the response regulator is in its phosphorylated state. In this sense, two-component systems are the predominant signal transduction system in bacteria, being used for the response to a diverse set of environmental signals (for a review on the physiological role of two-component systems, please, see [17]). Two-component systems have attracted the attention of bioinformatics researchers for the past years. The abundance of these systems makes them particularly suitable for genomics studies. Many bacteria have two-component systems, the majority of them possessing multiple of them. In addition, sensor domains that are unrelated to the sensor kinase domain and the response regulator domain are abundant within two-component systems, further increasing their complexity. Crosstalk between different signaling systems completes the signaling network (for an early study on the signaling network, please, see [18]).

Genomics studies involving two-component systems include sequence comparisons of a single two-component system (chemotaxis) across many different bacteria [19], sequence based structural classification of several response regulators across many genomes [20], and the development of new protocols to identify two-component systems in newly sequenced genomes [21]. Evolutionary studies identified recently evolved signaling molecules, indicating increased selective pressure upon the bacteria [22]. A new database, Sentra [23,24], includes many two-component systems, as well as other signaling proteins. It was hypothesized that a network of two-component systems might equip the bacteria with a rudimentary form of intelligence [25].

All of these studies use a comparative genomics approach to obtain structural and/or functional information. As the function of most of these two-component systems (exception chemotaxis) lies in gene regulation, functional genomics experiments have been performed for a small number of them [26,27]. The data set used for this study constitutes the most complete compilation of data on gene regulation by two-component systems that is currently available [16]. We will use an integrated approach to analyze this data set, combining functional or domain information with gene expression data. Throughout this manuscript, we will refer to the two-component system mutants as attributes and the log expression ratios between mutant and wild-type as attribute values.

The objective of the study is to find functional groups that are preferentially regulated by a specific set of two-component systems. Such information is of interest in understanding gene regulation. The objective is different from conventional clustering approaches, in which the actual gene clusters are in the foreground [2] and functional information may be used to improve clustering results [3,10,28]. Functional information has also been considered in the context of determining the significance of clustering results [29]. Our approach, in contrast, finds the significance of the relationship between the function and the differential expression. A related algorithm, using a subspace-based distance measure, has been discussed previously [30], and applied to cell cycle experiments in yeast.

This study is based on the perspective that groups of similar data points represent patterns in the data. Most clustering techniques implicitly use this concept and density-based techniques [31] are explicitly based on it. Pattern-based techniques have also been used to find differentially expressed genes [32,33]. While most algorithms use the full data set to find patterns, in this study we only consider the subset of data points that have a particular property of interest. Properties may either be functional designations, as provided through Gene Ontology (GO-terms [34]) or hidden Markov models for protein domains (HMMs [35]). If the subset shows an inhomogeneous distribution of data points, then we conclude that the property is related to the gene expression data set.

The objective of our study is, hence, slightly different from the goals of GSEA, where a main motivation is summarized by Efron & Tibshirani [36] as "By borrowing strength across the gene-set, there is potential for increased statistical power". Towards this goal, it is essential to correctly take into consideration that not just one test is performed, but multiple. The GSEA algorithm does so by controlling the false discovery rate [37]. In a multiple hypothesis testing context, overlap between gene sets has to be carefully taken into consideration [38,39]. In our work, the focus is on establishing that any one pattern we find is significant. It is not our intention to increase the significance of the gene expression experiment, but rather to find non-obvious patterns involving multiple, possibly independent, experiments. We thereby follow the pattern mining paradigm, which typically takes the perspective that any one reported pattern should be significant, but each pattern is an independently determined entity [40].

## Results and discussion

### Algorithm

**Figure 3:**
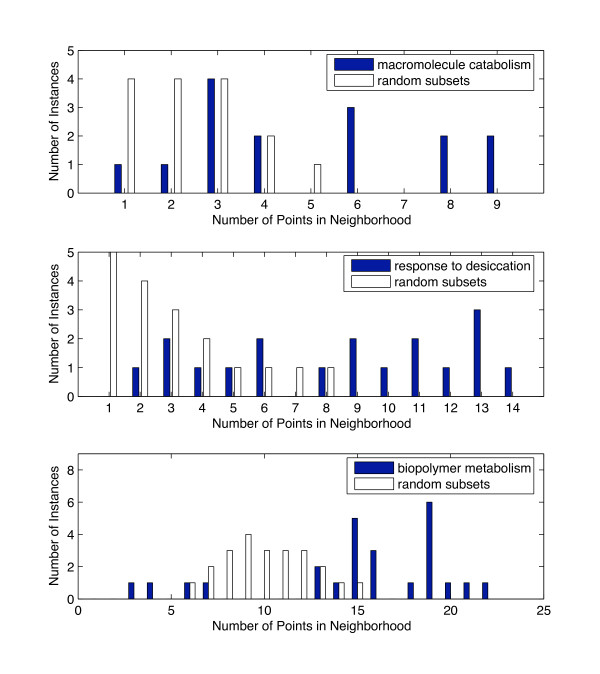
**Experimental and randomized histograms for the functions macromolecule catabolism, response to desiccation, and biopolymer metabolism**. A density measure that is evaluated for each data point is given by the number of neighbors that are close according to a product similarity measure. The blue bars in the histograms represent the sum of all these densities. Those vector pairs are expected to be most relevant that exhibit a large absolute value of differential expression and a small angle between vectors. The white bars show the randomly distributed vectors that are expected to have some neighbors as well. Subsets of genes that have the same number of elements as the protein function under consideration were randomly selected. The histogram was constructed for this random subset. The process was repeated multiple times and averaged over 20 runs. The top panel shows the histogram for the macromolecule catabolism function, the middle panels for the response to desiccation function, and the bottom panel for the biopolymer metabolism function.

**Figure 4:**
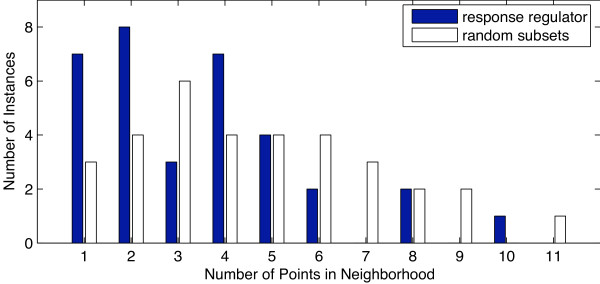
**Experimental and randomized histograms for the function response regulators**. Experimental and randomized histograms were constructed as described for Fig. 2. The response regulator function is one example of a function for which the experimental and the randomized histogram did not yield any major differences.

The algorithm has two objectives: (1) Identifying subsets that have a distribution that significantly differs from what would be expected for a random subset and (2) finding those data points that have more neighbors than expected. We define a density measure that is evaluated for each data point, and is given by the number of neighbors that are close according to a product similarity measure. Product similarity is used, rather than cosine similarity or Euclidean distance, since we expect those vector pairs to be most relevant that exhibit a large absolute value of differential expression as well as a small angle between vectors. The product similarity measure for vectors x^(*j*) ^and x^(*k*)^, with coordinates $x_{i}^{\left(j\right)}$ and $x_{i}^{\left(k\right)}$ respectively, is defined as follows

(1) $$\begin{array}{lll} S_{jk} & = & \sum_{i=1}^{d}x_{i}^{\left(j\right)}x_{i}^{\left(k\right)}\\ & & \begin{array}{ccc} 1\leq j\leq N & \text{and} & 1\leq k\leq N \end{array} \end{array}$$

where N is the number of selected data points, and *d *the number of dimensions, i.e. the number of experiments that are being considered. Vectors for which *S*_*jk *_exceeds a threshold *t *are considered neighbors. The threshold is given as

(2) $$\begin{array}{c} t=\mu d\\ \mu \in (0,1) \end{array}$$

where *μ *is chosen to be 0.3 in the evaluation. The algorithm does not strongly depend on the choice of *μ *as will be discussed in the section on choices within the algorithm. Each data point is associated with a density that is of type integer: the number of neighbors that satisfy the product similarity criterion. The occurring density values for all data points can be summarized using histograms. Density calculations are done on column-wise *z*-normalized data [41], i.e. for each attribute, the mean is subtracted and the attribute values are divided by the standard deviation. The rationale for using column-wise rather than row-wise normalization is that overall large absolute log expression ratios of individual genes are thereby preserved. This choice is also discussed in detail later. Fig. 3 and 4 show examples of histograms of the observed density values that are derived using the product similarity criterion (blue bars). These examples are derived as part of the evaluation on the Oshima data set on two-component systems [16], which is discussed in more detail in the next section.

Even randomly distributed vectors are expected to have some neighbors. We, therefore, have to evaluate the expected distribution of densities. This is done by randomly selecting a subset of genes that has the same number of elements as the protein function under consideration. A histogram is then constructed for the random subset. The process is repeated multiple times and the results averaged over 20 runs. Table 1 summarizes this resampling-based algorithm. Figs. 3 and 4 (white bars) show the distribution for random data sets in addition to the experimental ones.

**Table 1:** Resampling-based Algorithm

**Data**: *genes*;	/* expression values */
**Data**: *functions*	/* for each function */
**Result**: *significance, tailGenes*;	/* vector of zeros */
**1 ***normGenes *= normalize(*genes*);	
**2 ***hist *= zeros(1, nPts);	
**3 foreach ***f *∈ *function ***do**	
**4 ** *subset *= findPoints(*normGenes*, *f*);	
**5 ** **foreach ***x *∈ *subset ***do**	
**6 ** *dens *= NumberOfNeighbors(x);	
**7 ** *hist*(*dens*)++;	
**8 ** *randHist *= findRandomHistogram(1, *nPts, normGenes*);	
**9 ** *significance*(*f*) = chiSquaredGoodynessOfFit(*hist, randHist*);	
**10 ** *tailGenes*(*f*) = findTailGenes(*hist, randHist*);	
**11 return ***significance, tailGenes*	

An observed histogram is considered significant if it would be unlikely to encounter it based on a randomly selected set of genes. A *χ*^2 ^goodness-of-fit test is used to compare the histogram with its randomized counterpart. We consider patterns as significant, if the comparison yields a *p*-value ≤ 0.05. The methods section provides details on the significance testing. Fig. 3 shows three vector-item patterns that are considered significant, while Fig. 4 shows one counter example that is not considered significant.

### Application of the algorithm to two-component system data

**Figure 5:**
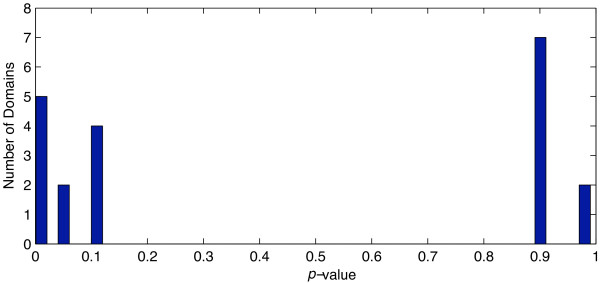
***p*-values for all domain sets**. *p*-values of the *χ*^2 ^goodness-of-fit test were performed on all 20 domains. Seven domains were considered significant.

We applied the algorithm to the two-component system data from Oshima and coworkers [16]. Throughout the entire data set, expression ratios represent the expression of mutants divided by those of the wild-type strain. Therefore, an expression ratio above 1 indicates that the gene is repressed by the corresponding two-component system. Expression ratios below 1 indicate activation by the two-component system. Log expression ratios for individual experiments are considered dimensions in a *d*-dimensional vector space, where *d *is the number of experiments considered. Histograms for all functional groups were derived. Seven functional designations were found to be significant, as shown in Table 2 and Fig. 5. The histograms for the gene ontology terms GO:0009057 = macromolecule_catabolism, GO:0009260 = response_to_desiccation, and GO:0043283 = biopolymer_metabolism are presented in Fig. 3.

**Table 2:** Significant vector-item patterns

**Function Name**	*p***-Value**	**Variance for group**	**Variance for tail genes**	**Number of genes in group**	**Number of genes in tail**
cellular biosynthesis	1.88e-008	0.025	0.028	37	11
macromolecule catabolism	0.011	0.033	0.055	15	7
carbohydrate metabolism	3.18e-7	0.029	0.039	44	15
cellular macromolecule metabolism	1.98e-8	0.025	0.030	32	12
macromolecule biosynthesis	1.08e-5	0.025	0.030	32	12
biopolymer metabolism	5.92e-5	0.025	0.030	34	13
response to desiccation	0.019	0.027	0.029	18	11

Column 1: Function name from GO annotation.

Column 2: *p*-value for standard *χ*^2 ^goodness-of-fit test.

Column 3: The average expression level of genes with the given function.

Column 4: The average expression level of genes with the given tail.

Column 5: Number of genes with the function.

Column 6: Number of genes in the tail.

Table 2 shows the *p*-values for each of the significant functional groups. The fifth column shows how many genes have the respective functional designation. When constructing histograms, some genes may have substantially more neighbors than would be expected from the randomized or theoretical model. These genes are considered particularly important since they have an unusual number of similar genes and may hence be considered as representing a pattern as was shown in Fig. 1. We define the tail of the histogram as those genes on the right side of the distribution, for which the expected density is less than 1. Table 2 shows in column 6 how many of the genes in each of the functional groups are tail genes. Notice that all significant patterns involve GO-terms and none of them Pfam HMMs. This observation is not surprising given that GO terms are expected to represent protein function far more effectively.

**Figure 6:**
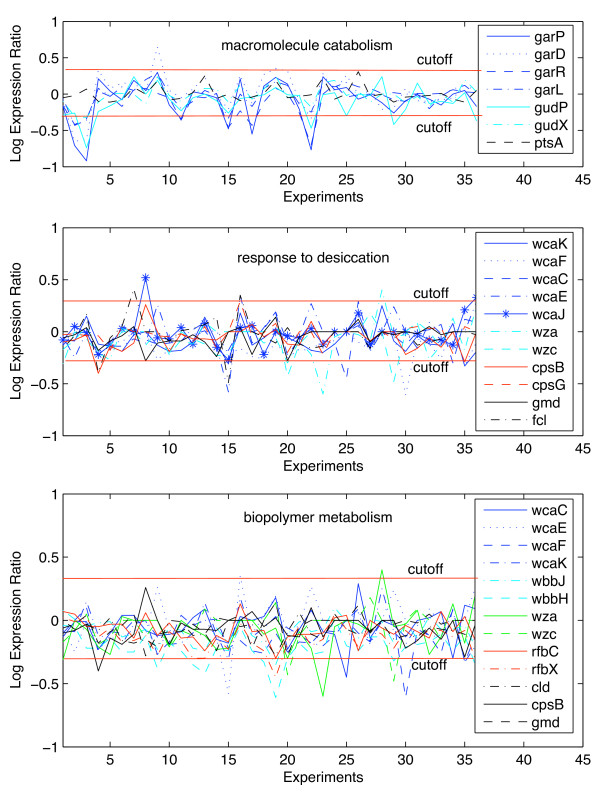
**Gene expression profiles for the functions macromolecule catabolism, response to desiccation, and biopolymer metabolism**. Profiles were obtained for all those genes in the functional groups of catabolism, response to desiccation, and biopolymer metabolism that have a larger number of points in the neighborhood than any of the points in the histograms for the random subset (blue tails in the histograms). The individual genes that form this group are indicated in the inserted legends. The numbers on the x-axis symbolize the individual two-component systems. The order of two-component systems (attributes) is identical to the original data set [16]. The following two-component systems are discussed in the text: #3 is OmpR/EnvZ, #4 is BasSR, #9 is YfhA, #15 is UvrY, #16 is YpdAB, #19 is DcuSR, #20 is NtrBC, and #22 is ArcB.

To understand our result better we also calculated the variance of the expression of genes that have the functional designation (column 3) in comparison with the overall data set (0.02676). We expect that the genes with a significant functional designation should rather be more clearly differentially expressed, i.e. have a higher variance of the differential expression. This is not required for our algorithm and the variance is not used in our algorithm. However, it might be an indication of a problem if significant functional designations were consistently less expressed. The same holds to an even greater extent for the genes that are in the tail of the distribution (column 4). These genes represent the patterns that contribute to the significance of the functional designation. If the patterns were due to genes that showed a low differential expression, the value of our observations to biologists would be questionable. In fact, when we used a previous algorithm [30] on our data set, this is exactly what we found: Significant patterns were found that were due to genes with an exceptionally low variance of differential expression. Later, we will present details on the comparison between our previous and our current algorithm and show that the variance of expression can be used as a means of validating if our results are useful from a biological perspective. Fig. 6 shows the expression profiles of all those genes in the above functional groups that have a larger number of points in the neighborhood than any of the points in the histograms for the random subset (blue tails in the histograms). The individual genes that form this group are indicated in the inserted legends. The numbers on the x-axis symbolize the individual two-component systems. The order of two-component systems (attributes) is identical to the original data set [16]. For the purpose of this study, #3 is OmpR/EnvZ, #4 is BasSR, #9 is YfhA, #15 is UvrY, #16 is YpdAB, #19 is DcuSR, #20 is NtrBC, and #22 is ArcB. These are the two-component systems for which the grouping of expression ratios is most visible within the profiles. Tables 3, 4, and 5 provide the log_10 _expression ratios for the genes that are in the histogram tails. The first row in both tables lists the two-component systems and the first column the genes that belong to the respective functional group. Log expression ratios that are < log_10_(0.5) or > log_10_(2) are presented in bold face.

**Table 3:** Macromolecule catabolism

	**OmpR/EnvZ**	**YfhA**	**UvrY**	**DcuSR**	**ArcB**	**Protein Function**
***garP***	**-0.92**	0.29	**-0.48**	0.22	**-0.77**	D-galactarate transporter
*garL*	**-0.37**	0.29	**-0.34**	0.17	-0.24	2-dehydro-3-desoxygalactarate aldolase
*garR*	**-0.74**	0.19	-0.27	0.09	**-0.42**	Tartronate semialdehyde reductase
***garD***	**-0.64**	**0.64**	-0.21	**0.35**	**-0.33**	D-galactarate dehydratase
***gudP***	**-0.74**	0.19	-0.27	-	**-0.42**	D-glucarate transporter
*gudX*	**-0.33**	0.24	-0.08	0.24	-0.18	D-glucarate dehydratase
*ptsA*	0.06	0.18	-	0.18	-0.02	Phosphotransferase system I

Column 1: Genes that are part of the function macromolecule catablism. The first gene in each operon is printed in bold.

Column 2 through 6: Two-component systems that are involved in the regulation of the genes in column 1. These are the most dramatic peaks in Fig. 6, top panel.

Column 7: Functions of the proteins that are encoded by the genes in column 1.

**Table 4:** Response to desiccation

	**BasSR**	**UvrY**	**NtrBC**	**YpdAB**	**Protein Function**
*wza*	-0.19	-0.22	-0.24	-	Outer membrane auxillary Wza
*wzc*	-0.19	-	**-0.43**	-	Autophosphorylating protein tyrosine kinase Wzc
*wcaC*	-0.24	-0.19	-0.22	0.03	Putative colonic acid biosynthesis glycosyl transferase
*wcaE*	-0.20	**-0.59**	-0.11	**0.34**	Putative colonic acid biosynthesis glycosyl transferase
*wcaF*	-0.25	-0.23	-0.25	-0.17	Putative colonic acid biosynthesis acetyl transferase
*gmd*	-0.19	-	-0.28	0.04	Fucose biosynthesis, GDP-D-mannose 4,6-dehydratase
*fcl*	**-0.40**	**-0.49**	0.04	**0.34**	NADPH dependent GDP-L-fucose synthase
*cpsB*	**-0.41**	0.00	-0.12	-	Mannose 6-phosphate isomerase
*cpsG*	-0.04	-	-0.21	**0.30**	Phosphomannomutase isozyme
*wcaJ*	-0.22	-0.28	-0.03	0.05	Putative colonic isozyme biosynthesis
					UDP-glucose lipid carrier transferase
*wcaK*	-0.12	**-0.32**	-0.01	0.17	Colanic acid biosynthesis protein

Column 1: Genes that are part of the function response to desiccation. The first gene in each operon is printed in bold.

Column 2 through 5: Two-component systems that are involved in the regulation of the genes in column 1. These are the most dramatic peaks in Fig. 6, middle panel.

Column 6: Functions of the proteins that are encoded by the genes in column 1.

**Table 5:** Biopolymer metabolism

	**BasSR**	**UvrY**	**NtrBC**	**YpdAB**	**DcuSR**	**Protein Function**
*wzb*	-0.10	-	0.05	-0.04	0.25	Protein tyrosine phosphatase
*wzc*	-0.19	-	**-0.43**	-	0.13	Autophosphorylating protein tyrosine kinase Wzc
*wcaC*	-0.24	-0.19	-0.22	0.03	-0.22	Putative colonic acid biosynthesis glycosyl transferase
*wcaE*	-0.20	**-0.59**	-0.11	**0.34**	-0.11	Putative colonic acid biosynthesis glycosyl transferase
*wcaF*	-0.25	-0.23	-0.25	-0.17	-0.25	Putative colonic acid biosynthesis acetyl transferase
*gmd*	-0.19	-	-0.28	0.04	-0.28	Fucose biosynthesis, GDP-D-mannose 4,6-dehydratase
*cpsB*	**-0.40**	0.00	-0.12	-	0.00	Mannose 6-phosphate isomerase
*wcaK*	-0.12	**-0.32**	-0.01	0.17	-0.07	Colanic acid biosynthesis protein
*rfbC*	-0.06	-0.17	-0.12	0.13	**-0.30**	D-TDP-4-dehydrorhamnose 3,5-epimerase
*rfbX*	-0.13	-0.24	-0.19	-0.03	**-0.46**	O-antigen translocase
*wbbH*	-0.22	-0.24	**-0.30**	-0.06	**-0.60**	O-antigen translocase
*wbbJ*	-0.03	-0.19	-0.12	0.00	**-0.36**	O-acetyltransferase
*cld*	-0.09	0.00	-0.10	-0.06	**-0.30**	Chain length regulator

Column 1: Genes that are part of the function biopolymer metabolism. The first gene in each operon is printed in bold.

Column 2 through 6: Two-component systems that are involved in the regulation of the genes in column 1. These are the most dramatic peaks in Fig. 6, bottom panel.

Column 7: Functions of the proteins that are encoded by the genes in column 1.

### Biological significance of the data

The first functional group of study is GO:0009057 = macromolecule_catabolism (Table 3). This group contains seven genes in four operons. Three operons include genes for galactarate and glucarate degradation. These are *garD*, *garP *(also containing *garLRK*), and *gudP *(also containing *gudD*). The fourth operon is *ptsA *(also containing *fsaB *and *gldA*) that encodes a phosphotransferase system.

The results suggest that two-component signaling plays an important role in the regulation of the galactarate and glucarate genes. This is important because little has previously been known about the regulation of these genes. Galactarate and glucarate are contained in various fruits. They can serve as growth substrates for several bacteria, including *E. coli*. The pathway that is used for the degradation of galactarate and glucarate leads to the production of pyruvate and glycerate.

The second functional group that is analyzed more closely is GO:0009269 = response_to_desiccation (Table 4). The eleven genes listed in Table 4 are all part of the *cps *operon. They encode enzymes, such as glycosyl and acetyl transferases and other auxiliary proteins that contribute to the formation of the colanic acid capsule (*cps*). The genes for the colanic acid capsule are clustered at about 45 min on the chromosome and expressed from a single promoter upstream of the gene *wza *[42]. This promoter is characterized by its -10 and -35 sites, as well as the RcsAB box that permits binding of RcsAB [43]. The Rcs system is the major system of regulation for the *cps *operon. It constitutes an unusual form of a phosphorelay, involving two of each, the kinase and the response regulator domains. It is not included in the data set that was used for this study [16].

The two-component systems that appear as most important for the regulation of the capsule genes (Table 4) respond to diverse environmental signals, such as iron [26], carbon [44], nitrogen [45], oxygen [46], and osmolarity [47]. It becomes obvious that the production of capsules is under tight environmental control, including many two-component systems. This regulation is in addition to the known regulation by the Rcs system [48]. It is consistent with the observation that colanic acid capsules have no known role in the virulence of the bacteria [49], but are needed for various lifestyles outside the host. Examples for stressful situations include osmotic shock or desiccation. Complex gene regulation by several two-component systems would enable the bacteria to perform this complex adaptation.

The third functional group under investigation is GO:0043283 = biopolymer_metabolism. As the most interesting observation, this group contains many of the genes of the *cps *operon that were discussed above. However, additional genes are included in this group. These are listed at the bottom of Table 5 and include *rfbC*, *rfbX*, *wbbH*, *wbbJ*, and *cld*. The regulation of these five genes by DcuSR is all above the two fold threshold and, therefore, more pronounced than regulation of any of the other genes by any of the other two-component systems. It appears that DcuSR is the major regulator of these genes. Additional minor regulators might be UvrY and NtrBC.

**Figure 7:**
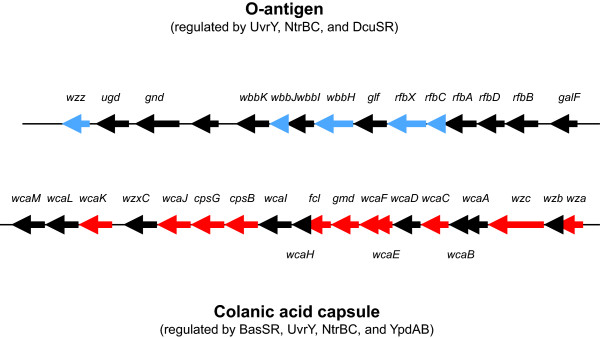
**Schematic of the capsule gene cluster**. Each arrow represents one gene in the direction of transcription. Genes in red are genes that are contained in the response to desiccation function. This operon encodes proteins of the colonic acid capsule. Genes in blue are genes that are contained in the biopolymer metabolism function and are not included in the response to desiccation function. This operon encodes components of the O-antigen. Two-component systems that regulate each group of genes are indicated.

The *rfbC*, *rfbX*, *wbbH*, *wbbJ*, and *cld *genes are all involved in the synthesis of the O-antigen. The O-antigen is another surface polymer (for a review on capsular polysaccharides in *E. coli*, please, see [50]). Variation in its structure is the reason for the many O-specific serotypes that *E. coli *can exhibit. It provides a challenge to the host immune system, having to continuously adapt to new bacterial surface proteins. The colanic acid genes and the O-antigen genes cluster together on the *E. coli *chromosome, with the O-antigen genes being located downstream of the capsule genes (Fig. 7). An additional promoter (besides the *wza *promoter) has been postulated within this gene cluster. It resides in the intergenic region between *galF *and *rfbB *and probably constitutes a transcriptional start site for the O-antigen genes. According to our data, the *wza *promoter could be regulated by BasSR, UvrY, NtrBC, and YpdAB. The *rfbB *promoter might be regulated by UvrY, NtrBC, and DcuSR. This indicates that our algorithm is able to predict transcriptional units within gene clusters, based upon similarity in function and gene expression profiles.

### Biological questions and hypotheses derived from this study

We used the new analysis of the previously published data set [16] to design biological questions that could lead to future experiments. Three questions were asked in particular:

1. Under which conditions is *E. coli *able to use galactarate and glucarate as growth substrates? The observation that several two-component systems are involved in the regulation of the galactarate and glucarate genes indicates that *E. coli *might be able to grow on galactarate and glucarate under more environmental conditions than previously thought.

2. Are the two-component systems BasS/BasR, BarA/UvrY, NtrB/NtrC, and ArcB/ArcA involved in the formation of biofilms? In a recent review article, we summarized a network of regulation that involved the agellar master regulator FlhD/FlhC and several two-component systems [51]. The network affected the expression and synthesis of several cell surface organelles, including capsules. The formation of biofilms was used as a connecting theme to explain regulations within the network. This study extends previous observations. The number of two-component systems that is involved in the regulation of biofilms might be larger than anticipated earlier [51]. Whether any or all of these two-component systems are really involved in the formation of biofilms, can easily be determined experimentally.

3. Do our data indicate new functions for two-component systems of previously unknown function? YfhA is the response regulator that is phosphorylated by its cognate kinase, YfhK [52]. Considering that the expression ratios for YfhA are higher than for the other two-component systems that contribute to the regulation of the galactare and glucarate genes (Table 3), one might assume that YfhA is a major regulator of these genes. YpdAB is another two-component system whose function is currently unknown. It appears in our data as the major negative regulator of the colanic acid capsule genes (Table 4).

### Testing of one hypothesis derived from this study

**Figure 8:**
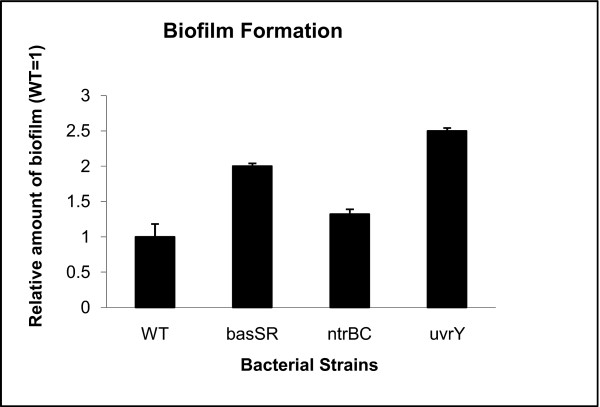
**Quantitative biofilm assay**. The ability to form biofilms was compared between wild-type bacteria and mutants in *basSR, ntrBC*, and *uvrY*. Bioluminescence is indicative of biomass and calculated relative to wild-type *E. coli*. The experiment was performed three times. Average and standard deviations are presented.

We tested part of hypothesis two, the involvement of two-component systems in biofilm formation, with a quantitative biofilm assay that was previously described [53]. The two-component systems tested were BasSR, NtrBC, and UvrY. Mutants in *basSR, ntrBC*, and *uvrY *were compared to their isogenic wild-type strain (Fig. 8). All three mutants produced more biofilm than the wild-type. This is consistent with our hypothesis.

### Application of the algorithm to a second data set

**Figure 9:**
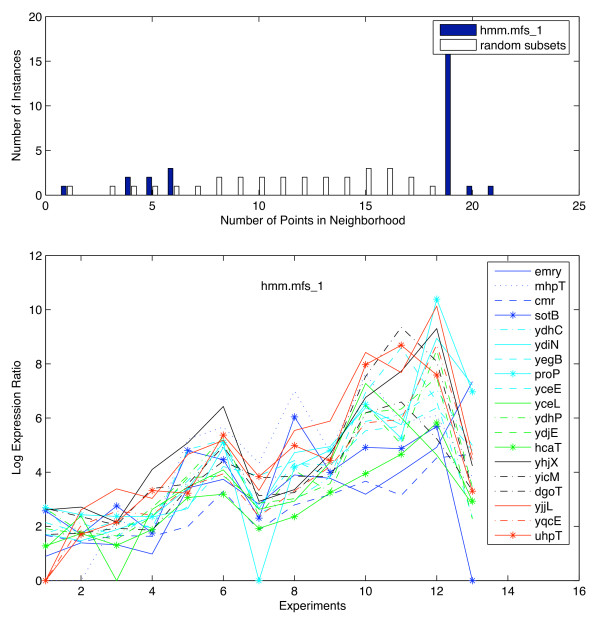
**Experimental and randomized histogram and profile for the function hmm.mfs_1 for the Baev data set**. Top panel: A density measure that is evaluated for each data point is given by the number of neighbors that are close according to a product similarity measure. The blue bars in the histograms represent the sum of all these densities. Those vector pairs are expected to be most relevant that exhibit a large absolute value of differential expression and a small angle between vectors. The white bars show the randomly distributed vectors that are expected to have some neighbors as well. Subsets of genes that have the same number of elements as the protein function under consideration were randomly selected. The histogram was constructed for this random subset. The process was repeated multiple times and averaged over 20 runs. Bottom panel: Gene expression profile for the function hmm.mfs_1. Profiles were obtained for all those genes in the functional group of hmm.mfs_1 that have a larger number of points in the neighborhood than any of the points in the histograms for the random subset (tails in the histograms). The individual genes that form this group are indicated in the inserted figure legends. The numbers on the x-axis symbolize the individual two-component systems. The order of two-component systems (attributes) is identical to Fig. 5.

To test the algorithm for its general usefulness, we applied it to a second data set. The data set by Baev and coworkers [54-56] contains a total of 12 experiments, each representing a time point in the growth profile of *E. coli *growing in LB at 37°C. Applying the algorithm to this data set yielded one structural group of proteins that exhibited similar expression profiles. Histograms and profiles are shown in Fig. 9. The group hmm.mfs-1 consists of transporters that belong to the major facilitator superfamily. Many of them are involved in drug export and multidrug resistance. This functional group was not analyzed in the three previous publications [54-56]. This analysis is a good example of how the biological question asked and the algorithm used impact the results that are to be expected. Baev and coworkers identified functional groups first and analyzed their expression profiles in a second step. They found proteins transporting a certain compound and the enzymes that are used for the degradation of this compound exhibit similar expression profiles. Our algorithm identifies patterns involving functional groups and gene expression data in one step. We found one large group of transporters. This demonstrates that applying a new algorithm to an already well analyzed data set can still yield new information.

### Comparison with the GSEA algorithm

We compared our algorithm with the gene set enrichment analysis algorithm, GSEA [6]. In order to use this algorithm, the gene expression data were transformed to GSEA format, then phenotype files as well as gene sets for each domain were created. For both the Oshima and Baev data sets, no domain was considered significantly enriched at nominal *p*-value *<*0.05 by the GSEA algorithm. All possible combinations of parameters, which include 'metric for ranking genes' and 'gene list sorting mode', were used.

### Comparison with clustering and biclustering followed by enrichment analysis

We then applied biclustering to the Oshima data set. For this comparison, we first considered the Bimax algorithm [57], which is available as part of the Biclustering Analysis Toolbox BicAT [58]. With default settings, this algorithm did not return any results. When setting the 'discretization threshold' to 0.3, which approximately corresponds to the log_10_(2) we received 950 clusters as a result, but each of them with no more than 4 genes which was too small for further statistical analysis.

We then used the Expander software [9], following the suggestions in the documentation (providing unnormalized log_2 _values of the expression ratios as input, followed by a standardization of "Mean 0 and Variance 1", i.e. row-wise *z*-normalization). For consistency reasons we used the same gene annotation data as for our own algorithm. TANGO enrichment analysis was performed, both on the results of the SAMBA biclustering [4] and the CLICK clustering algorithm [59], using default parameters throughout. Both SAMBA and CLICK results led to the identification of two enriched functional groups, however only one of them (GO:0008610 = lipid_biosynthesis) was found by both algorithms. The SAMBA result showed enrichment for GO:0009269 = response_to_desiccation and the CLICK result for GO:0044260 = cellular_macromolecule_metabolism.

**Figure 10:**
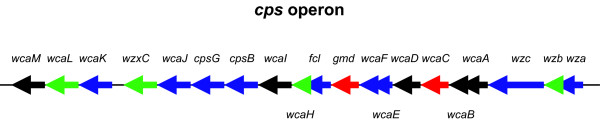
**Comparison of the two-step approach with our algorithm for GO:0009269 = response_to_desiccation**. Expander's [9] biclustering algorithm Samba followed by enrichment analysis Tango is used as exemplary two-step approach and compared with our single-step approach. Each arrow represents one gene in the *cps *gene cluster. The genes of the GO:0009269 = response_to_desiccation that were identified as significant are highlighted. Genes that were returned by both algorithm are highlighted in red, additional genes that were found by our algorithm are printed in blue and genes that were found only by the comparison algorithm are in green.

Both GO:0009269 = response_to_desiccation and GO:0044260 = cellular_macromolecule_metabolism are also found by our algorithm. In addition, our algorithm identifies five functional categories as significant that are not found by the comparison methods. As expected, based on the discussion that accompanies Fig. 2, our algorithm is able to identify patterns that are not accessible by a two-step method. Note that GO:0008610 = lipid_biosynthesis, which is found by both comparison methods does not satisfy the filter condition of a minimum of 15 genes that we applied to the data set when using our own algorithm. The clustering- and biclustering-based approaches also return a smaller number of genes as members of the significant clusters (4–8 genes). Fig. 10 highlights genes identified by each algorithm for GO:0009269 = response_to_desiccation. Two genes in the *cps *operon were considered significant by both ours and the SAMBA-based algorithm. Our algorithm found nine additional genes within the same functional group, whereas SAMBA/TANGO found only four additional genes. Both algorithms pointed to the same operon, in which all genes are expected to be similarly expressed and have related functions.

### Comparison with a previously proposed subspace-based algorithm

The development of this new algorithm was motivated by problems that we observed when applying one of our previous algorithms [30] (which we will call subspace-based) to the Oshima data set. The subspace-based algorithm calculates histograms based on the following definition of a neighborhood: A gene is considered a neighbor of another gene if it is within a predefined range for at least a predefined fraction of dimensions. That means that two genes can be considered neighbors because they both show a particularly small differential expression. Table 6 illustrates this problem: For the subspace-base algorithm (column 4), the variance of the differential expression of genes in the tail of the distribution is smaller than the variance of all genes with the respective functional designation. That means that the corresponding functional annotations are significant, based on genes that show little or no differential expression. Note that from a statistical perspective such functional annotations may very well be significant, but from a practical perspective they are only of interest to biological researchers if the goal is to identify housekeeping genes and not differentially expressed genes.

**Table 6:** Comparison of variance of gene expression level

**Function**	**Variance for tail genes**			**Variance for group**	**Variance overall**
			
	**Product similarity**		**Subspace algorithm **[30]		
				
	**Column-wise**	**Row-wise**			
cellular biosynthesis	0.028	0.023	0.018	0.025	0.027
macromolecule catabolism	0.055	0.044	not significant	0.033	
carbohydrate metabolism	0.039	0.025	0.017	0.029	
cellular macromolecule metabolism	0.030	0.022	0.023	0.025	
macromolecule biosynthesis	0.030	0.022	0.020	0.025	
biopolymer metabolism	0.030	0.023	0.018	0.025	
response to desiccation	0.029	0.020	not significant	0.027	
ABC transporter	not significant	0.028	not significant	0.026	

Column 1: Function name from GO annotation.

Column 2: The average expression level of genes in the tail for the product-based similarity. using column-wise *z*-normalization.

Column 3: The average expression level of genes in the tail for the product-based similarity. using row-wise *z*-normalization.

Column 4: The average expression level of genes in the tail for the subspace-based similarity.

Column 5: The average expression level of genes with the function.

Column 6: The average expression level of genes in the whole data set.

The similarity measure used in this work is inherently designed to measure the degree to which positive or negative differential expression matches between any two genes. Table 6 illustrates that for the new algorithm, as expected, the variance in the tail is indeed larger than the overall variance (column 2). Note that the variance of the differential expression does not enter the algorithm as such but rather is a result.

### Choices within the algorithm

We tested how sensitive the algorithm is with respect to the value of the threshold value *μ*. We compared the outcome of the analysis for eight different choices of *μ *in the range from 0.03 to 0.39. Six of the 20 functional annotations were significantly related to the gene expression data for all choices of *μ*, and 3 were not significant for any of the choices. For each of the remaining 4 choices, the value at *μ *= 0.3 matched the result for at least half of the parameter choices. It can be concluded that the algorithm is not very sensitive to the choice of *μ*.

In addition, we evaluated the impact of data imputation on the results. Our data set has 14% unavailable data, and hence it can be argued that selecting those from the estimated distribution of values is more appropriate than replacing them with the mean. We used the multiple imputation software by Allison [60] for this purpose. The functional groups, for which the majority of *μ*-values indicate a significant relationship with the gene expression data, as well as the result for *μ *= 0.3 remain the same. We found that no more than, on average, one gene is considered as being in the tail without imputation and not in the tail with imputation or vice versa for the functional annotations that are significant in both settings. Hence, we conclude that imputation does not have a strong impact on the outcome of the analysis either. Finally, we tested how strongly the normalization affects the result. For this comparison, we applied *z*-normalization to rows and then applied the algorithm as previously. We found that only one additional functional annotation was considered significant, the ABC_transporter domain from Pfam. We also checked whether the significant relationships were due to highly expressed genes. The results are shown as column 3 of Table 6. It can be seen that the variance of expression values for the genes in the tail is typically smaller than the corresponding quantity based on all genes that share the functional annotation. Only two of the annotations show higher variance in the tails (ABC_transporter and macromolecule_catabolism). For the column-wise normalization that we use otherwise, the genes in the tail of the distribution have an higher variance for all functional annotations, i.e. the genes in the tail are more clearly differentially expressed. We, therefore, consider the results with column-wise normalization to more likely represent useful information.

### Performance

**Figure 11:**
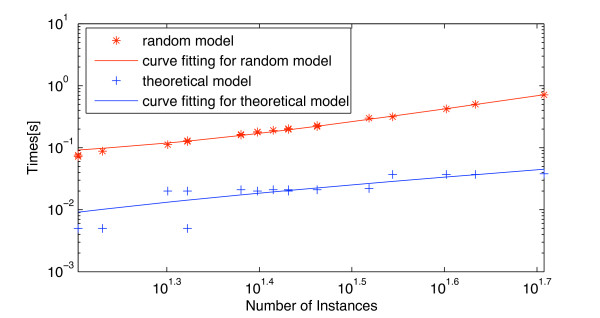
**Runtime for evaluation of all significant domains**. Comparison of complexity between random model and theoretical model. The complexity of the theoretical model is significantly smaller. The slope of the logarithmic regression is 1.90 for the random model and 1.83 for the theoretical model.

The algorithm scales linearly with the number of domain or function subsets. For each domain, scaling as a function of domain size is approximately quadratic, as Fig. 11 shows. For the *E. coli *data set that was used in this study, the quadratic complexity is not a problem, and it can be seen that execution times are so small that performance is not expected to be a bottleneck even for larger genomes.

The runtime for one subset can be expressed as follow:

(3) $$T_{c}=(R+1)*(T_{extract}+T_{hist})+T_{\chi^{2}-test}$$

Where *T*_*c *_is the runtime of the algorithm for one subset, *T*_*hist *_the time to create the histogram for one subset, *T*_*extract *_is the time for extracting the subset of genes, *R *is the number of runs for random subsets (*R *= 20 in the evaluation) and $T_{\chi^{2}-test}$ is the time for the statistical analysis. Under the assumption that data sets are small enough such that all genes can be kept in memory, the time is dominated by *T*_*hist*_, which is quadratic in the number genes in a subset.

Notice that the main contribution to *T*_*c *_comes from the histogram evaluation on random subsets. In the following section, we discuss a theoretical model for deriving the random histogram without resampling. The model ignores correlations that may occur even among unrelated genes, and is hence not expected to be as accurate as the randomized evaluation. Nevertheless, it provides an alternative if the data sets are very large.

#### Theoretical model for histograms

The resampling version of the algorithm, which is used for the remainder of this study, is robust with respect to uctuations in the data set. The theoretical derivation of a comparison distribution that is presented in this section is given for the sake of performance improvement where necessary, but is not expected to lead to equally robust results. For the random model that is used in the theoretical derivation, we assume that all experimental data follow a normal distribution. This assumption is not expected to be fully accurate, since gene expression experiments typically do not exactly follow such a distribution. The calculation also assumes that dimensions for the random comparison model are unrelated, which is a different approximation. Both assumptions only apply to the theoretical model and not to the resampling approach that is used for this study. The resampling model is expected to be substantially more accurate, and the theoretical model should only be used if the computational complexity of the resampling model is considered prohibitive.

The coordinates of two experiments are denoted by vectors **x **and **y**. All those pairs of genes are considered significantly related, for which the product is greater than threshold *t*. The expected probability that the product for any two experiments is beyond the threshold *t *can be calculated by integrating the following expression over the relevant Gaussian distribution functions:

(4) $$p=\theta \left(\sum_{i}x_{i}y_{i}-t\right)$$

where *θ *is the Heaviside step function, which is 1 for a positive argument and 0 otherwise. We integrate over all directions of **x **and **y **with their respective weights

(5) $$\int \theta \left(\sum_{i}x_{i}y_{i}-t\right){\left(\frac{1}{\sqrt{2\pi }}\right)}^{2n}e^{-\frac{x^{2}}{2}}e^{-\frac{y^{2}}{2}}{\text{d}}^{n}x{\text{d}}^{n}y$$

Note the data are normalized using *z*-normalization, resulting in mean 0 and standard deviation 1 for both vectors **x **and **y**. The radius of the vector **x **will be denoted by *r *and the integration re-written

(6) $$\int {\left(\frac{1}{\sqrt{2\pi }}\right)}^{n}e^{-\frac{x^{2}}{2}}{\text{d}}^{n}x=\int S_{n}r^{n-1}e^{-\frac{r^{2}}{2}}\text{d}r$$

where *S*_*n *_is the surface of a hypervolume in *n *dimensions

(7) $$S_{n}=\{\begin{array}{ll} \frac{2^{\frac{n+1}{2}}\pi^{\frac{n-1}{2}}}{(n-2)!!} & \text{for }n\text{ odd}\\ \frac{2\pi^{\frac{n}{2}}}{\left(\frac{n}{2}-1\right)!} & \text{for }n\text{ even} \end{array}$$

The integration over **y **can be written as an integration over the coordinate in the direction of **x**, which we will denote as *z *and the vector perpendicular to **x**, represented by **u**. We can now rewrite the *θ*-function as follows

(8) $$\theta \left(\sum_{i}x_{i}y_{i}-t\right)=\theta (rz-t)$$

This function does not depend on **u**. The *n *- 1-dimensional integration over **u**, therefore only has a normalized *n *- 1 dimensional Gaussian function as integral, and thereby trivially gives the result 1. The probability of a product beyond the threshold is

(9) $$p=\int \int \frac{1}{{\left(\sqrt{2\pi }\right)}^{n}}e^{-\frac{r^{2}}{2}}S_{n}r^{n-1}\theta (rz-t)e^{-\frac{z^{2}}{2}}\text{d}r\text{ d}z$$

The integration over z can be performed by recognizing that the *θ *function is 1 only for $z>\frac{t}{r}$ and 0 otherwise.

(10) $$p=\int \frac{1}{2{\left(\sqrt{2\pi }\right)}^{n-1}}S_{n}r^{n-1}e^{-\frac{r^{2}}{2}}\left[1-\text{erf}\left(\frac{t}{\sqrt{2}r}\right)\right]\text{d}r$$

Given this probability *p*, we can calculate the theoretical distribution for the selected subsets:

(11) $$h_{k}=N\left(\begin{array}{c} N\\ k \end{array}\right)p^{k}{\left(1-p\right)}^{N-k}$$

**Figure 12:**
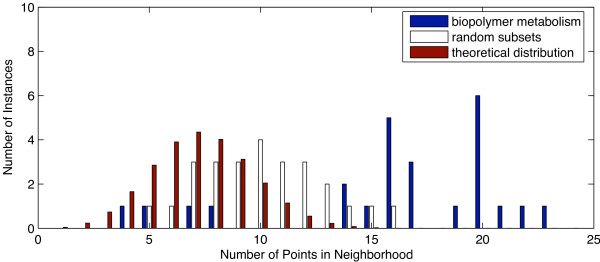
**Resampled and theoretical histograms for the macromolecule catabolism function**. In addition to the histogram in Fig. 2A, the histogram is presented that resembles the theoretical distribution of genes.

Fig. 12 shows the histograms of the theoretical distribution, resampled distribution (random subsets) and the observed distribution (biopolymer metabolism) for one of the three discussed functions. The resampled distribution is slightly more stretched than the theoretical one, which can be attributed to correlations among the experiments that are not considered in the theoretical model. Fig. 11 shows that the complexity of the algorithm is significantly decreased, although it is still roughly quadratic.

Using the theoretical model, the algorithm of Table 1 can be modified as shown in Table 7.

**Table 7:** Distribution-based Algorithm

**Data**: *genes*;	/* expression values */
**Data**: *functions*	/* for each function */
**Result**: *significance, tailGenes*;	/* vector of zeros */
**1 ***normGenes *= normalize(*genes*);	
**2 ***hist *= zeros(1, nPts);	
**3 foreach ***f *∈ *function ***do**	
**4 ** *subset *= findPoints(*normGenes*,*f*);	
**5 ** **foreach ***x *∈ *subset ***do**	
**6 ** *dens *= NumberOfNeighbors(x);	
**7 ** *hist*(*dens*)++;	
**8 ** **if ***NunmberOf(genes) greater than a threshold ***then**	
**9 ** *randHist *= findTheoreticalHistogram(1, *nPts, normGenes*);	
**10 ** **else**	
**11 ** *randHist *= findRandomHistogram(1, *nPts, normGenes*);	
**12 ** *significance*(*f*) = chiSquaredGoodnessOfFit(*hist, randHist*);	
**13 ** *tailGenes*(*f*) = findTailGenes(*hist, randHist*);	
**14 return ***significance, tailGenes*	

## Conclusion

We have introduced an algorithm that permits relating protein functions to gene expression data. It allows us to identify functions that are common in proteins whose genes are regulated similarly across the spectrum of two-component systems. Our analysis led to the development of biological hypotheses that suggest further experimentation. Initial experiments confirmed one of the hypotheses.

## Methods

The data set used for this study was constructed by Oshima and coworkers [16]. They examined mRNA levels in 36 two-component deletion mutants and compared them to those of wild-type bacteria. Growth conditions were kept constant between experiments. The data were expressed as expression ratios, dividing the expression level of each gene in the mutant by that of the wild-type. The mutant collection covers all of the two-component systems that *E. coli *possesses. In cases where kinase and response regulator are encoded by genes that form one operon, this two-component system only yields one mutant. In other cases, kinase and response regulator genes are far apart on the chromosome and then there are two mutants to cover these two genes.

As a first processing step, the data were converted to log expression ratios by taking a log_10_. We then applied the *z*-normalization that is required by the algorithm itself. About 14% of the data points are missing in the whole data set. This can happen because not all genes are expressed under all conditions. We replaced the missing values with a log ratio of 0, since 0 does not contribute to the similarity using the product measure. As a next step, we eliminated genes that were not differentially expressed, i.e. we only kept those genes that had an absolute log expression ratio of at least log_10_(2) for at least one of the two-component systems. 2570 genes satisfied this criterion and were used for the remainder of the analysis.

As function data we used the GO and PF annotations from previously published work [61], and a threshold was applied that requires an annotation to be held by at least 15 genes, leaving us with 13 functions. A standard *χ*^2 ^test was used on the histograms after the following preprocessing: Bins at both ends of the distribution were merged until the expected number was at least 5. If the intermediate bins had an expected number smaller than 5, then pairs of bins were merged until no more bins had an expected number smaller than 5. A function was considered as significantly related to the expression data if the *χ*^2 ^goodness-of-fit test yielded a *p*-value ≤ 0.05. The algorithm was implemented in C++, compiled by C++Builder 6.0.

A quantitative biofilm assay was used to test one of the hypotheses that our algorithm had generated. This assay involved the measurement of ATP, an energy molecule whose concentration is considered consistent across various growth conditions [62], in a bioluminescence reaction. The assay was performed as previously described [53] with 12 wells per strain on a 96 well plate. Triplicate experiments were performed, average and standard deviation are presented. The bacterial strains used were BW25311 [63,64], as well as their isogenic *basSR, ntrBC*, and *uvrY *mutants [65]. These strains are the same strains that the data (Tables 4 and 5) had been derived from. Biofilms were formed in tryptone broth (1% tryptone, 0.5% NaCl) at 37°C for 40 h.

## Competing interests

The authors declare that they have no competing interests.

## Authors' contributions

JW and AMD developed the algorithm and MKT and BMP performed the biological analysis of the data. PS performed the biofilm experiments. The manuscript was jointly written and approved by all authors.
